# PatchCTG: A Patch Cardiotocography Transformer for Antepartum Fetal Health Monitoring

**DOI:** 10.3390/s25092650

**Published:** 2025-04-22

**Authors:** M. Jaleed Khan, Manu Vatish, Gabriel Davis Jones

**Affiliations:** Oxford Digital Health Labs, Nuffield Department of Women’s & Reproductive Health (NDWRH), University of Oxford, Women’s Centre (Level 3), John Radcliffe Hospital, Oxford OX3 9DU, UK; jaleed.khan@wrh.ox.ac.uk (M.J.K.); manu.vatish@wrh.ox.ac.uk (M.V.)

**Keywords:** antepartum, cardiotocography, fetal health, transformer, biomedical time series

## Abstract

Antepartum Cardiotocography (CTG) is a biomedical sensing technology widely used for fetal health monitoring. While the visual interpretation of CTG traces is highly subjective, with the inter-observer agreement as low as 29% and a false positive rate of approximately 60%, the Dawes–Redman system provides an automated approach to fetal well-being assessments. However, it is primarily designed to rule out adverse outcomes rather than detect them, resulting in a high specificity (90.7%) but low sensitivity (18.2%) in identifying fetal distress. This paper introduces PatchCTG, an AI-enabled biomedical time series transformer for CTG analysis. It employs patch-based tokenisation, instance normalisation, and channel-independent processing to capture essential local and global temporal dependencies within CTG signals. PatchCTG was evaluated on the Oxford Maternity (OXMAT) dataset, which comprises over 20,000 high-quality CTG traces from diverse clinical outcomes, after applying the inclusion and exclusion criteria. With extensive hyperparameter optimisation, PatchCTG achieved an AUC of 0.77, with a specificity of 88% and sensitivity of 57% at Youden’s index threshold, demonstrating its adaptability to various clinical needs. Its robust performance across varying temporal thresholds highlights its potential for both real-time and retrospective analysis in sensor-driven fetal monitoring. Testing across varying temporal thresholds showcased it robust predictive performance, particularly with finetuning on data closer to delivery, achieving a sensitivity of 52% and specificity of 88% for near-delivery cases. These findings suggest the potential of PatchCTG to enhance clinical decision-making in antepartum care by providing a sensor-based, AI-driven, objective tool for reliable fetal health assessment.

## 1. Introduction

Antepartum Cardiotocography (CTG) plays a pivotal role in fetal health monitoring, serving as a critical assessment tool in prenatal care. By using ultrasound-based techniques to record the Fetal Heart Rate (FHR) and uterine activity, CTG provides clinicians with data on fetal well-being through the examination of heart rate variability and response patterns to uterine contractions. Uterine contractions are measured using a tocodynamometer (TOCO). Established methods like the Dawes–Redman (DR) computerised CTG system [1] offer valuable criteria for interpreting CTG patterns, enhancing clinical decisions that help mitigate the risks of adverse outcomes such as neonatal acidaemia, hypoxia, and stillbirth [2]. Despite its widespread adoption in clinical settings, visual CTG analysis suffers from high intra- and inter-observer variability. Studies indicate that clinicians fail to identify key FHR patterns in up to 35–92% of cases [3,4], with an inter-observer agreement as low as 29% and a false positive rate of approximately 60% [5,6]. The Dawes–Redman algorithm, developed to provide an objective, rule-based interpretation of CTG traces, helps mitigate some of its limitations. However, it is primarily designed to confirm fetal well-being rather than detect adverse outcomes, leading to high specificity (90.7%) but poor sensitivity (18.2%) in identifying fetal distress [7]. These limitations underscore the need for more advanced AI-driven models capable of incorporating pregnancy-specific risk factors and providing more reliable prognostic assessments.

Recent advancements in artificial intelligence and machine learning, particularly in deep learning, have demonstrated considerable potential in automating and improving the accuracy of CTG interpretation. By leveraging deep learning models, researchers have advanced the detection of adverse outcomes in CTG signals through feature extraction, noise reduction, and classification tasks, providing more consistent assessments than manual interpretation [8]. Transformers excel at handling sequential data, including biomedical time series, due to their ability to capture complex temporal dependencies by dynamically learning correlations across input elements, which makes them highly promising for CTG analysis [9]. With self-attention mechanisms, transformers capture complex temporal dependencies in CTG data, focusing on relevant segments of FHR and uterine activity patterns. Despite these advancements, several challenges persist in applying deep learning to CTG-based fetal health monitoring. Existing models fall short in capturing the physiological responses in CTG, largely due to signal variability across patients, monitoring conditions, and clinical contexts [10]. Issues like insufficient data diversity, high computational costs, and a lack of generalizability across different clinical settings often limit the performance of deep learning models on CTG data. Addressing these challenges requires specialised models that can adapt to different temporal patterns while maintaining a robust performance.

In this paper, we introduce the Patch Cardiotocography Transformer (PatchCTG), a patch-based transformer model designed to classify adverse and normal outcomes in antepartum CTG recordings reliably. The PatchCTG model builds on recent advancements in patch-based transformers for time series [11], which demonstrate promising performances in sequence compression and feature representation by segmenting signals into patches. Unlike traditional CTG analysis approaches, PatchCTG applies instance normalisation and channel-independent processing to manage distribution shifts and capture the distinct temporal dynamics of the FHR and uterine activity. By leveraging patch-based tokenisation and self-attention, PatchCTG provides enhanced computational efficiency and adaptability to longer temporal windows, making it well suited to CTG data, where signal length and variability pose significant modelling challenges. We rigorously evaluated PatchCTG and benchmarked it against the DR algorithm and deep learning methods on a subset of the extensive Oxford Maternity (OXMAT) dataset [12], which includes over 20,000 CTG traces from diverse clinical outcomes. While data acquisition challenges, such as signal noise, missing values, and device-related inconsistencies, are common in CTG datasets, the OXMAT dataset mitigates these issues through rigorous preprocessing, quality control, and clinician-reviewed curation that spans over three decades. This ensures that PatchCTG is trained and validated on a dataset with minimal acquisition-related biases.

The main contributions of this study are as follows:

- We introduce PatchCTG, a transformer-based architecture tailored for CTG signals through patch-based segmentation, instance normalisation, and channel-independent processing. This design effectively captures both local and global temporal dependencies by segmenting signals into patches, mitigating distribution shifts through instance normalisation, and allowing the separate modelling of the FHR and uterine contraction channels. These architectural choices address the non-stationarity of CTG data, enabling the more accurate classification of adverse and normal outcomes.
- We employed the Optuna hyperparameter optimisation framework [13] to identify the best configuration of PatchCTG for fetal health classification. This systematic approach finetunes model depth, attention heads, embedding dimensions, dropout rates, and other key parameters, ensuring that PatchCTG achieves a high predictive performance while maintaining its generalizability across CTG samples and cohort variations.
- The detailed experimental results seen with the OXMAT dataset demonstrate the promising performance of PatchCTG across various classification thresholds and time windows relative to delivery. PatchCTG achieved an AUC of 0.77, with a specificity of 88% and sensitivity of 57% at Youden’s index threshold, demonstrating its adaptability to different clinical conditions. Our experimentation involved cohort balancing, sequence standardisation, and multiple temporal thresholds to ensure model robustness and clinical applicability.
- We benchmarked the performance of PatchCTG against the traditional Dawes–Redman algorithm [1] and an optimised hybrid deep learning model, showing that PatchCTG outperforms both. Compared to Dawes–Redman (AUC 0.67) and the CNN-LSTM-Transformer model (AUC 0.73), PatchCTG (AUC 0.77) demonstrated a superior ability to capture temporal dependencies while achieving a well-balanced trade-off between sensitivity and specificity (52% and 88% for near-delivery cases), making it a strong candidate for real-world fetal monitoring applications.

The remainder of this paper is structured as follows: Section 2 reviews related work on antepartum fetal monitoring using machine learning. Section 3 presents the proposed PatchCTG model architecture and Section 4 presents the experimental setup and the results, including dataset preprocessing, hyperparameter optimisation, performance evaluation, and benchmark comparison. Section 5 discusses the experimental results of PatchCTG, its potential impact on clinical practice, and future directions for its development and is followed by the conclusion in Section 6.

## 2. Related Work

The Dawes–Redman (DR) system has long served as the gold standard in electronic fetal monitoring, providing a rule-based algorithm for interpreting CTG signals by analysing FHR variability and responses to uterine contractions [1]. However, it is primarily designed to confirm fetal well-being rather than predict adverse outcomes, leading to a high specificity (90.7%) but very low sensitivity (18.2%) [7]. In contrast, visual CTG interpretation remains highly subjective, with inter-observer agreement as low as 29% and a false positive rate of approximately 60%, often leading to unnecessary interventions [3,4,5,6]. These limitations highlight the need for more advanced AI-driven models that can enhance predictive accuracy while reducing observer bias. Neppelenbroek et al. [14] further underscored this issue, showing that only professionals with high training consistency in controlled environments achieved satisfactory inter- and intra-observer agreement, which varied significantly, from 64% to 98%. Jones et al. [7] evaluated the performance of the DR algorithm in predicting adverse outcomes, using 4196 antepartum FHR recordings and excluding those with incomplete data or terminated analyses. Their findings indicated that while the DR algorithm showed high sensitivity (91.7%) in detecting fetal well-being, its specificity for adverse outcomes was low (15.6%), limiting its predictive utility in high-risk pregnancies.

To mitigate the subjectivity and variability of manual CTG assessments, machine learning approaches have been proposed as alternatives that can improve reliability and interpretative accuracy. Traditional ML methods such as the EMD-SVM model by Krupa et al. [15] employed Empirical Mode Decomposition (EMD) for feature extraction and Support Vector Machine (SVM) classification, achieving 87% accuracy and a high agreement (kappa value 0.923) with expert evaluations. Georgieva et al. [16] employed an ensemble of Artificial Neural Networks (ANNs) for adverse outcome prediction, achieving a sensitivity of 60.3% and specificity of 67.5%. Fei et al. [17] developed an Adaptive Neuro-Fuzzy Inference System (FCM-ANFIS) that achieved 96.39% accuracy, outperforming conventional classifiers. Chen et al. [18] introduced a Deep Forest model that handled overlapping normal and suspicious classifications with 92.64% accuracy on the UCI dataset [19]. However, while these traditional approaches demonstrated some promise, their limited feature extraction capabilities often restricted their generalisation across diverse datasets.

With advancements in deep learning, more sophisticated models like Convolutional Neural Networks (CNNs) and Long Short-Term Memory (LSTM) networks have gained traction in CTG analysis, allowing for improved temporal feature extraction and pattern recognition within FHR variability. Petrozziello et al. [20] used Multimodal CNNs to predict fetal distress by processing FHR and uterine contraction data, achieving a True Positive Rate (TPR) of 53% and a 15% false positive rate (FPR). Ogasawara et al. [21] proposed CTG-net, a three-layer CNN model that achieved an AUC of 0.73 ± 0.04, outperforming SVM and k-means clustering. Xiao et al. [22] used a multiscale CNN-BiLSTM model to capture spatial and temporal features, reaching a sensitivity of 61.97% and specificity of 73.82% on the CTU-UHB dataset [23]. Fei et al. [24] developed a Multimodal Bidirectional Gated Recurrent Unit (MBiGRU) network, achieving an AUC of 0.93%. Although these DL approaches showed an improved ability to extract intricate temporal patterns, their capacity to capture long-range dependencies in non-stationary CTG data and to generalise was still limited.

In recent years, hybrid models have emerged, which integrate diverse neural architectures to harness their complementary strengths. For instance, Spairani et al. [25] combined a Multi-Layer Perceptron (MLP) and CNN for mixed quantitative and image-derived inputs, achieving an accuracy of 80.1% but showing limited sensitivity. Feng et al. [26] used an ensemble of SVM, eXtreme Gradient Boosting (XGB), and random forest models, reaching 0.9539 accuracy on the UCI dataset. Zhang et al. [27] developed DT-CTNet, which combined an XGBoost ensemble and CNN-based tracking, achieving a diagnostic accuracy of 96.3%. Chen et al. [28] introduced an Unsupervised Domain Adaptation (UDA) model, DANNMCTG, to handle cross-device discrepancies, achieving an accuracy of 71.25%. These models have demonstrated the potential for enhanced generalisation and interpretability; however, their performance frequently depends on extensive feature engineering and high computational demands.

The emergence of self-attention mechanisms and transformer-based models has shifted the landscape in time series classification, with an enhanced capacity to capture complex temporal dependencies. In terms of CTG data, self-attention models have shown the potential to address the limitations of CNNs and RNNs by dynamically focusing on the relevant parts of the signal. Asfaw et al. [29] introduced a Gated Convolutional Multi-Head Attention (GCMHA) model, which combined CNNs with attention mechanisms to refine temporal dependencies, achieving a sensitivity of 49.08% at a 15% FPR. Wu et al. [30] proposed the Ensemble Transformer-Convolutional Neural Network (ETCNN), designed to capture both short- and long-term features by segmenting FHR patterns into acceleration and deceleration phases. The ETCNN demonstrated improved segmentation accuracy, achieving an 80.68% accuracy for accelerations and a 78.24% accuracy for decelerations.

Beyond general transformer-based models, emerging research [11] suggests that patch-based transformers offer unique advantages for time series analysis. By segmenting sequences into patches, these models reduce input dimensionality while preserving local and global temporal information, improving feature extraction and computational efficiency. Patch-based segmentation has enhanced computational efficiency and improved feature representation in complex, quasi-periodic or non-stationary data, making it a promising approach for CTG classification. The design of patch-based transformers aligns well with CTG data requirements, where signal length and variability are considerable challenges for conventional approaches.

## 3. Proposed Method

**Figure 1:**
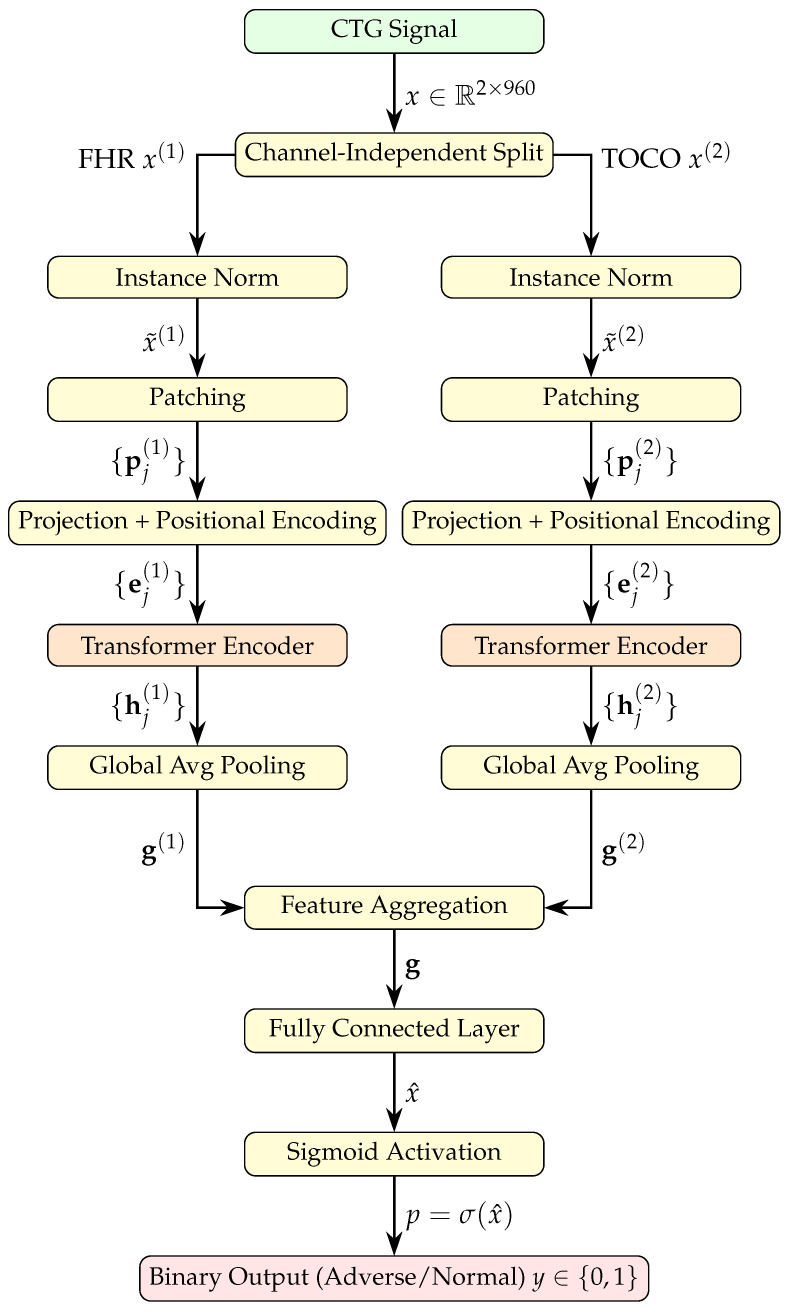
PatchCTG architecture for antepartum CTG classification. The input CTG signal, consisting of FHR and TOCO channels, is processed through separate paths comprising instance normalisation, patch embedding, and channel-independent transformer encoders. The resulting pooled representations are aggregated and passed through a classification head with a sigmoid output to predict binary outcomes.

The Patch Cardiotocography Transformer (PatchCTG) is a transformer-based architecture designed to classify CTG signals into binary outcomes: adverse or normal. It builds upon the time series forecasting transformer architecture [11] and has been adapted for the specific task of CTG classification. PatchCTG focuses on time series classification using FHR and TOCO signals, with each consisting of L=960 time steps (corresponding to one hour of recording). PatchCTG efficiently extracts temporal dependencies from CTG signals through a workflow that includes instance normalisation for mitigating distribution shifts; patching for sequence compression; channel-independent processing; a transformer backbone for temporal modelling; and a classification head with pooling, dense layers, and sigmoid activation for prediction. The PatchCTG architecture is illustrated in Figure 1.

In time series analyses, particularly in clinical datasets, the characteristics of input signals can vary significantly due to various factors, such as variations between patients and recording conditions. To mitigate the resulting distribution shift effects in the training and testing data, PatchCTG adopts **instance normalisation**, which has proven effective in reducing such distribution issues [31,32]. Instance normalisation independently standardises each univariate channel (FHR or TOCO) to have no mean or unit variance. It recalculates mean and variance statistics for each sequence during inference, which helps to reduce patient-to-patient variability and ensures robustness across various recording conditions. Specifically, each univariate channel (FHR or TOCO) is independently standardised to have no mean or unit variance. Given an input time series $x^{\left(i\right)}=\left(x_{1}^{\left(i\right)},x_{2}^{\left(i\right)},\ldots ,x_{L}^{\left(i\right)}\right)$, the normalised version ${\tilde{x}}^{\left(i\right)}$ is computed as follows:

(1) $${\tilde{x}}^{\left(i\right)}=\frac{x^{\left(i\right)}-\mu^{\left(i\right)}}{\sigma^{\left(i\right)}},$$

where $\mu^{\left(i\right)}$ and $\sigma^{\left(i\right)}$ are the mean and standard deviation of $x^{\left(i\right)}$, respectively. This ensures that the model is robust to scale variations and more stable during training, which is essential for effective learning from clinical data, which often involve different baselines for each patient and varied signal characteristics.

The PatchCTG method adopts a patching mechanism, which segments each univariate signal into a sequence of patches, inspired by the success of patch-based strategies in time series forecasting [11,33]. Patching effectively captures both local and global temporal trends, reduces input sequence length, and facilitates smoother temporal transitions in medical time series where gradual physiological changes occur. Given an input univariate signal $x^{\left(i\right)}$, the patching mechanism divides $x^{\left(i\right)}$ into non-overlapping or overlapping patches of a fixed length *P*. Specifically, a patch $p_{j}^{\left(i\right)}$ of the signal is defined as

(2) $$p_{j}^{\left(i\right)}=\left(x_{jS+1}^{\left(i\right)},x_{jS+2}^{\left(i\right)},\ldots ,x_{jS+P}^{\left(i\right)}\right),\;j=0,1,\ldots ,N-1,$$

where *S* is the stride (step size), *P* is the patch length, and *N* is the total number of patches and is given by

(3) $$N=\left\lfloor \frac{L-P}{S}\right\rfloor +1.$$

The stride *S* controls the overlap between consecutive patches. By adjusting the stride, PatchCTG can create overlapping patches (S<P) to capture smoother transitions across temporal segments or non-overlapping patches (S=P) to focus on distinct episodes. Patching reduces the input sequence length by representing each patch as a single token, thereby enhancing computational efficiency. Each patch also retains local semantic information, which is critical for understanding physiological trends and events, such as identifying patterns in FHRs that correlate with uterine contractions.

PatchCTG employs a **channel-independent transformer encoder** for each univariate signal (FHR or TOCO). By processing each channel independently, the model learns the unique temporal dynamics of each physiological signal before combining them for classification. The input encoding begins by projecting each patch from its original input space into a higher-dimensional latent space using a linear transformation:

(4) $$z_{j}^{\left(i\right)}=W_{P}p_{j}^{\left(i\right)},\;W_{P}\in R^{P\times d},$$

where *d* is the latent dimensionality of the patch representation. To preserve temporal information, positional encodings $W_{\mathrm{pos}}\in R^{N\times d}$ are added to each patch representation, resulting in

(5) $$e_{j}^{\left(i\right)}=z_{j}^{\left(i\right)}+W_{\mathrm{pos}}^{\left(j\right)},$$

where $e_{j}^{\left(i\right)}$ represents the patch encoded with temporal information. This positional encoding ensures that the model can learn to interpret the sequential changes in CTG signals, which is important for understanding FHR decelerations or accelerations in response to uterine contractions.

The **transformer backbone** consists of *L* encoder layers, each comprising two sub-layers:

- Multi-Head Self-Attention (MHSA): The multi-head self-attention mechanism enables PatchCTG to learn relationships between different patches within a given signal, providing a comprehensive view of temporal dependencies across the time series [33]. Given query, key, and value matrices Q, K, and V, the attention output A is computed as follows:
  (6) $$\mathrm{Attention}\left(Q,K,V\right)=\mathrm{softmax}\left(\frac{QK^{T}}{\sqrt{d_{k}}}\right)V,$$
  where $d_{k}$ is the dimensionality of the key vectors, and the scaling factor $\frac{1}{\sqrt{d_{k}}}$ ensures numerical stability.
- Feed-Forward Network (FFN): The feed-forward network is applied to each output of the MHSA. It consists of two linear transformations with a non-linearity in between (Gaussian Error Linear Unit, GELU). Given an input vector $h_{i}$, the FFN output $f_{i}$ is given by
  (7) $$f_{i}=\mathrm{GELU}\left(W_{1}h_{i}+b_{1}\right)W_{2}+b_{2},$$
  where $W_{1}$ and $W_{2}$ are learnable weight matrices and $b_{1}$ and $b_{2}$ are biases. Residual connections and layer normalization are employed to stabilize training and facilitate gradient flow across multiple layers.

PatchCTG handles missing values using a masking mechanism, which prevents the model from learning spurious relationships from incomplete data during attention computations. After processing the input patches through the transformer backbone, PatchCTG applies **global average pooling** across the time dimension. Given the output representations ${\left\{e_{j}^{\left(i\right)}\right\}}_{j=1}^{N}$, global average pooling computes

(8) $$g^{\left(i\right)}=\frac{1}{N}\sum_{j=1}^{N}e_{j}^{\left(i\right)},$$

where $g^{\left(i\right)}$ is the aggregated feature representation for each input sequence. By using global pooling, PatchCTG extracts meaningful summary statistics across the entire time horizon of each channel, capturing short-term variations and long-term trends, both of which may have clinical relevance in determining adverse outcomes. The pooled representation is then passed to a **classification head** consisting of a dense layer and a sigmoid activation function, which maps the aggregated features to a single output value:

(9) $$y^{\left(i\right)}=\sigma \left(W_{c}g^{\left(i\right)}+b_{c}\right),$$

where $W_{c}$ and $b_{c}$ are learnable parameters and σ(·) is the sigmoid activation function. The output $y^{\left(i\right)}$ represents the probability that the input CTG corresponds to an adverse outcome, with a threshold of 0.5 used to assign a binary class label (adverse or normal). The use of the global average pooling layer ensures that the final classification is informed by the entire temporal trajectory of each CTG signal, rather than focusing only on specific patches. This design is particularly important in medical time series analyses, where both long-term trends and short-term fluctuations can be indicative of clinical outcomes.

The training objective for PatchCTG is formulated as a **binary cross-entropy loss** function, which is suitable for this binary classification problem. Given the predicted probability $\hat{y}$ and the true class label y∈{0,1}, the binary cross-entropy loss L is defined as

(10) $$L=-\left(y\log\left(\hat{y}\right)+\left(1-y\right)\log\left(1-\hat{y}\right)\right).$$

The model parameters are optimised to minimise this loss across the training data.

## 4. Experiments and Results

### 4.1. Data Preprocessing and Organisation

The dataset used in this study was sourced from the Oxford Maternity (OXMAT) dataset [12], a comprehensive repository of CTG traces and maternal–neonatal health records collected from the Oxford University Hospitals maternity database at John Radcliffe Hospital. The OXMAT dataset contains over 211,000 CTGs, collected from more than 250,000 pregnancies between January 1991 and February 2024. Alongside CTG signals, the dataset includes over 250 clinical variables, which cover a range of maternal and neonatal outcomes such as Apgar scores, cord blood gas (CBG) values, birthweights, delivery types, medications, and other related health parameters. For the development of PatchCTG, we adopted the dataset preprocessing methodology described in [34] for cohort development and outcome categorisation. Raw digital CTG traces were extracted from singleton pregnancies between gestational weeks $37^{+0}$ and $41^{+6}$. The preprocessing involved removing CTG traces that were missing more than 30% of their signal information or had aborted a Dawes–Redman analysis before evaluation. Only traces that had undergone a successful Dawes–Redman analysis were included. Additionally, given that CTG signal quality can vary due to differences in acquisition devices, maternal factors, and gestational conditions, OXMAT [12] applies stringent signal validation, noise reduction techniques, and cross-hospital standardisation. This ensures that PatchCTG was evaluated on high-fidelity, clinically validated data, reducing acquisition-related artefacts that could bias its learning.

To establish the Adverse Pregnancy Outcome (APO) cohort, traces acquired within the 7 days prior to delivery were selected to ensure that the CTG patterns used for classification were temporally related to the outcome. The adverse pregnancy outcomes considered included acidaemia, stillbirth, asphyxia, extended Special Care Baby Unit (SCBU) admission, Hypoxic–Ischaemic Encephalopathy (HIE), a low Apgar score, and neonatal resuscitation at delivery. These outcomes were chosen based on their clinical significance and correlation with neonatal health risks. To develop the Normal Pregnancy Outcome (NPO) cohort, inclusion and exclusion criteria were applied to identify traces with positive outcomes. Pregnancies in the NPO cohort included liveborn singleton babies with a gestational age between $37^{+0}$ and $41^{+6}$ weeks, normal umbilical cord blood gas measurements, acceptable Apgar scores, and no major complications (e.g., emergency caesarean section or neonatal resuscitation). Traces from the NPO cohort were then matched to those of the APO cohort using one-to-one propensity score matching, controlling for key factors such as gestational age, maternal age, BMI, fetal sex, parity, and monitoring time prior to delivery.

After applying the inclusion and exclusion criteria and performing propensity score matching, we obtained a cohort consisting of 19,462 CTG traces (9731 in the NPO group and 9731 in the APO group). An 80–20 split was applied to this cohort for training and validation purposes. Additional preprocessing was performed to prepare the CTG signals for modelling. The FHR signal was adjusted to a range of [50,250] beats per minute, while the uterine contraction (TOCO) signal was adjusted to a range of [0,100], keeping −1 as an indicator of missing values. Both signals were subsequently normalised to a range of [0.0,1.0] to ensure a uniform input scale for the neural network. CTG signals of variable lengths were standardised to a fixed one-hour duration consisting of L=960 time steps. Longer CTG traces were segmented into 60-min windows, while shorter traces were padded to ensure a consistent input length. This standardisation facilitated efficient model training and ensured all input data had the same temporal length.

The final preprocessed dataset used to train and validate PatchCTG consisted of 20,589 CTGs, including 10,890 NPO traces (controls) and 9699 APO traces (cases). The 80–20 split resulted in 16,471 traces for training and 4118 traces for validation and testing. The balanced cohorts, achieved through propensity score matching, ensured that the training and validation sets were free from significant biases, with Standardised Mean Differences (SMDs) of less than 0.10 for all controlled factors. This cohort balancing step is crucial for enabling robust performance evaluation and minimising the risk of confounding factors during model training and testing.

### 4.2. Hyperparameter Optimisation

The performance of a deep learning model heavily depends on the appropriate selection of its hyperparameters. Therefore, a comprehensive hyperparameter optimisation process was conducted to determine the best configuration of the PatchCTG model for accurately classifying CTG signals into binary outcomes (adverse or normal) in terms of the Area Under the Curve (AUC). The hyperparameter optimisation aimed to enhance model generalizability while mitigating overfitting, with the objective of maximizing the AUC validation metric. We employed the Optuna hyperparameter optimisation framework [13] to perform an efficient and systematic search across a wide hyperparameter space. The optimisation process was formulated as a Bayesian optimisation problem, allowing us to iteratively explore the search space and focus on promising hyperparameter combinations based on previous trials. The goal was to identify the best hyperparameters that achieve the highest AUC score on the validation set, ensuring the reliable binary classification of adverse pregnancy outcomes. The hyperparameter search covered various components of the PatchCTG architecture, including the following:

- Transformer Encoder Layers: The number of encoder layers was varied from 3 to 6 to determine the model depth that most effectively captures temporal dependencies without leading to overfitting.
- Attention Heads: The number of attention heads was tuned using the set {4,8,16,32} to evaluate the impact of multi-head attention mechanisms on capturing complex temporal relationships.
- Model Dimensions: The embedding dimensionality was tuned using the set {64,128,192,256,384,512,640}, where higher dimensionality allowed for richer feature representations while lower dimensionality reduced the computational cost.
- Feed-Forward Layer Dimension: The hidden layer dimensionality within the feed-forward network was adjusted using the set {128,192,256,320,384,512,640} to balance the expressiveness and complexity of the model.
- Dropout Rates: Dropout rates for different components (transformer layers, fully connected layers, and attention heads) were tuned in the range of [0.1,0.5] to control overfitting and improve model robustness.
- Learning Rate: The learning rate was selected from the set $\left\{1\times 10^{-6},\;5\times 10^{-6},\;1\times 10^{-5},\;5\times 10^{-5},\;1\times 10^{-4},\;5\times 10^{-4},\;1\times 10^{-3}\right\}$ to identify the most suitable rate for efficient convergence of the model.
- Batch Size: The batch size was varied within the range {16,32,48,64} to determine the optimal trade-off between convergence stability and computational efficiency.
- Patching Parameters: The patch length and stride for sequence patching were tuned within the sets {4,8,16,32} and {4,8,16}, respectively, to explore different levels of sequence compression and overlapping temporal regions.
- Activation Function: Activation functions {ReLU,GELU,ELU} were evaluated to determine which non-linearity yielded the most expressive feature representations for the CTG data.

We conducted hyperparameter tuning over 100 trials using Optuna, with each trial representing a unique combination of hyperparameters. Each trial trained the model for a maximum of 60 epochs, with early stopping employed to halt training if no improvement was observed in the validation AUC for 10 consecutive epochs. This approach ensured computational efficiency while avoiding overfitting. The validation set, consisting of 20% of the preprocessed dataset, was used to evaluate the model’s performance during each trial, with the AUC score serving as the primary evaluation metric. After performing the hyperparameter optimisation, the following hyperparameters yielded the highest validation AUC of 0.77:

- Number of Encoder Layers: 6.
- Number of Attention Heads: 4.
- Model Dimension: 512.
- Feed-forward Layer Dimension: 128.
- Dropout Rate: 0.1.
- Fully Connected Layer Dropout: 0.4.
- Attention Head Dropout: 0.2.
- Patch Length: 16.
- Stride: 16.
- Kernel Size: 15.
- Activation Function: ReLU.
- Batch Size: 48.
- Learning Rate: $1\times 10^{-4}$.

The hyperparameter set identified highlights the importance of model depth, patch length, and dropout rates in achieving high performance. Specifically, the use of six encoder layers, a modest number of attention heads, and regularisation through dropout were key factors contributing to the effective learning of temporal dependencies without overfitting, thereby improving the model’s ability to generalise across different CTG signals.

### 4.3. Training, Finetuning, and Testing

The PatchCTG model underwent a comprehensive training, finetuning, and evaluation procedure to assess its effectiveness in classifying CTG signals into the APO and NPO classes. The process involved training on a large, balanced dataset, finetuning on specific subsets, and assessing its generalizability and performance stability across different configurations and cohorts. The performance of PatchCTG was primarily evaluated using the Area Under the Curve (AUC) metric, along with other classification metrics such as sensitivity, specificity, Positive Predictive Value (PPV), Negative Predictive Value (NPV), F1 score, and accuracy.

The initial training phase involved the entire dataset, which consists of 20,589 CTGs (10,890 NPOs and 9699 APOs), and used the hyperparameters that were optimised through the Optuna framework. The PatchCTG model was trained for a total of 50 epochs, with an early stopping criterion based on the validation AUC to ensure that the model generalised well without overfitting. The training–validation split was carried out at an 80–20 ratio, providing 16,471 samples for training and 4118 for validation and testing.

**Figure 2:**
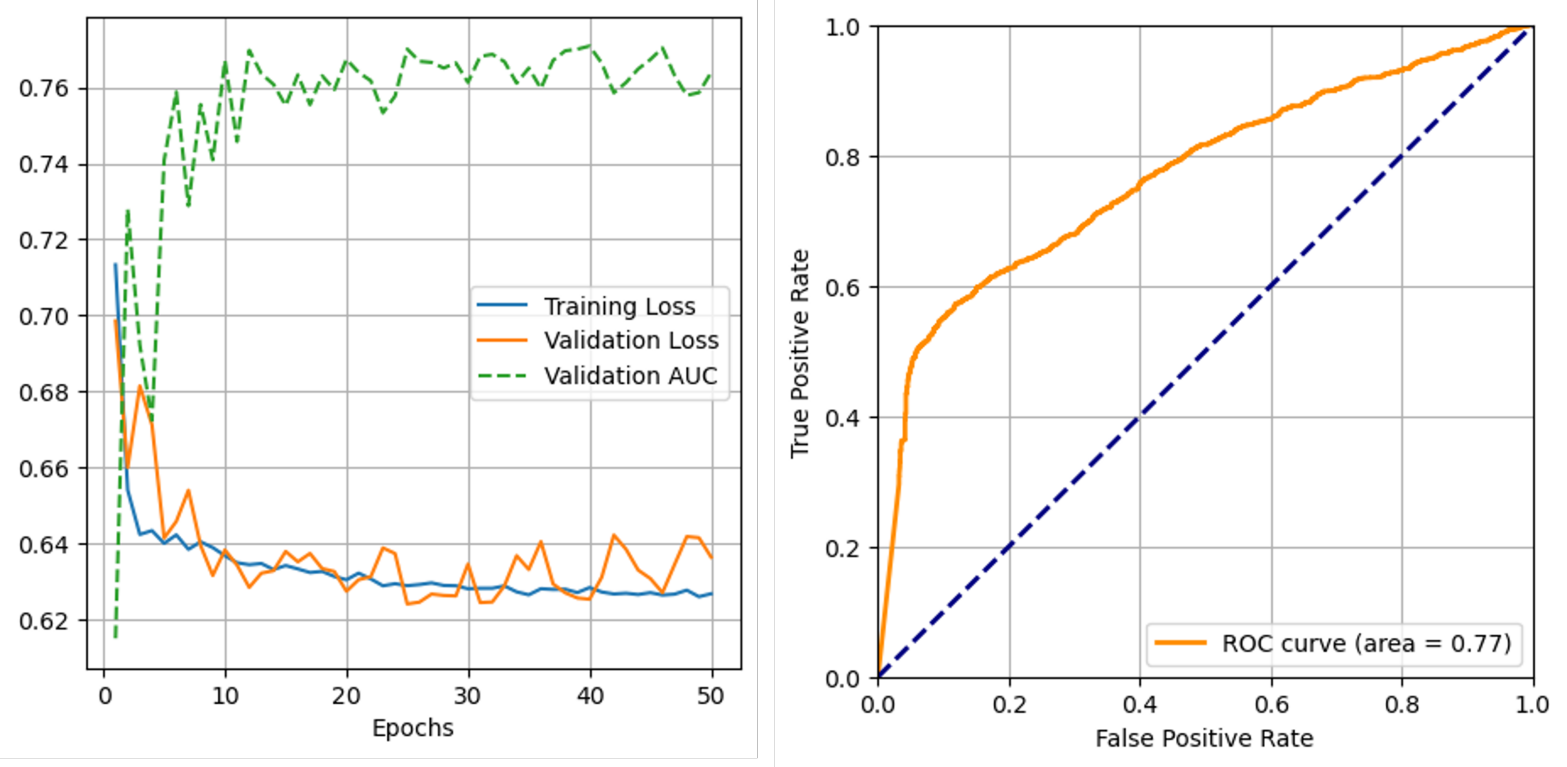
Training and validation results of the PatchCTG model on the complete dataset with optimised hyperparameters, showing convergence to approximately 0.77 AUC and consistent model performance throughout training. The orange line represents the ROC curve (model performance), and the blue dashed line represents random chance (baseline).

**Figure 3:**
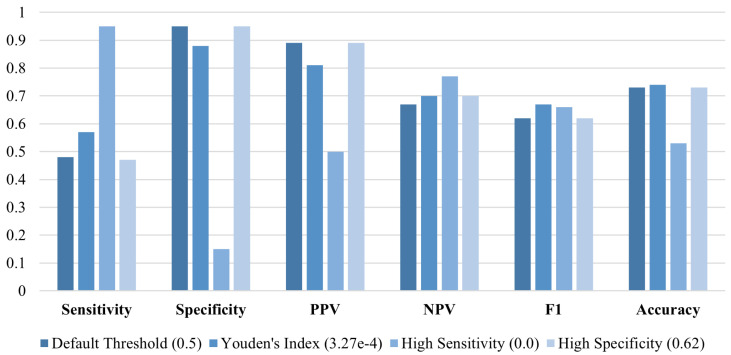
Performance of PatchCTG model trained and tested on the complete dataset on cases recorded up to 7 days prior to delivery, evaluated using sensitivity, specificity, PPV, NPV, F1 score, and accuracy for different classification thresholds.

Figure 2 presents the training and validation progress plots, showing the convergence of the model to an AUC of approximately 0.77. The ROC curve (Figure 2) demonstrates the ability of PatchCTG to distinguish between adverse and normal outcomes, with its AUC well above that of a random guess. The performance metrics obtained for different classification thresholds, including the default threshold, Youden’s index threshold, high sensitivity threshold, and high specificity threshold, are presented in Figure 3. These results indicate a well-balanced trade-off between sensitivity and specificity, depending on the threshold selected. At Youden’s index threshold, PatchCTG achieved a sensitivity of 57%, specificity of 88%, PPV of 81%, and an F1 score of 67%, which highlights the robustness of the model for clinical decision support.

**Figure 4:**
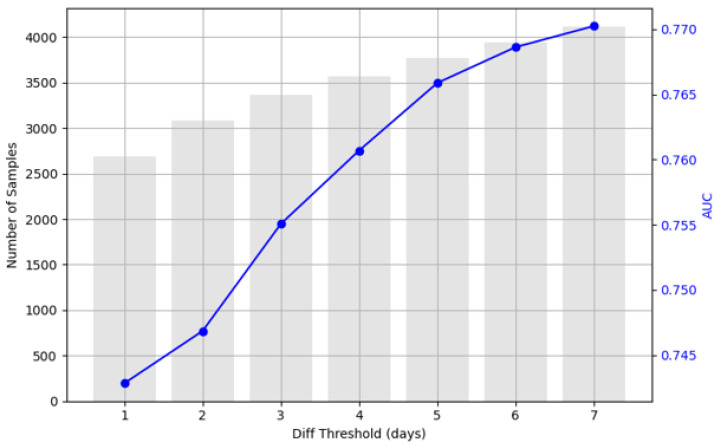
The performance of PatchCTG in terms of its AUC with varying thresholds of days to delivery (1–7) for the APO cohort, indicating a gradual improvement in the AUC as the temporal threshold expands.

To evaluate the impact of the temporal gap between the CTG recording and delivery outcome, the PatchCTG model was assessed using varying thresholds of days to delivery for the APO cohort. Specifically, the model was evaluated on subsets of the dataset that contained APO cases recorded within 1 to 7 days before delivery (Figure 4). The AUC increased from approximately 0.74 to 0.77 as the threshold increased from 1 to 7 days before delivery. The results indicated that CTG signals collected closer to delivery had a slightly lower predictive power compared to those recorded over a longer duration preceding the delivery. This could be due to increased variability and abrupt changes in physiological patterns closer to delivery, which might be more challenging for the model to predict accurately.

**Figure 5:**
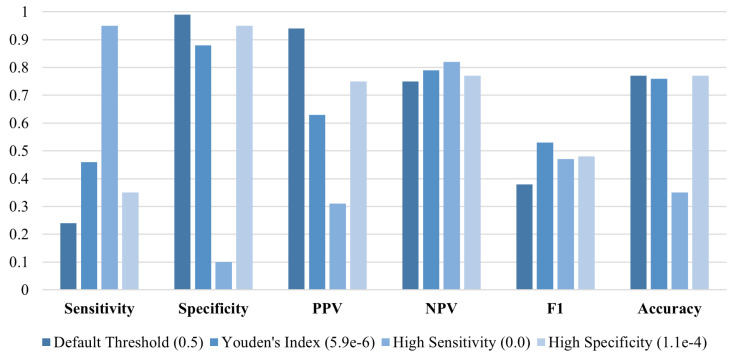
Results of PatchCTG model trained on a subset of data that included cases recorded 3–7 days prior to delivery and tested on a subset that included cases recorded up to 2 days prior to delivery (AUC = 0.73).

**Figure 6:**
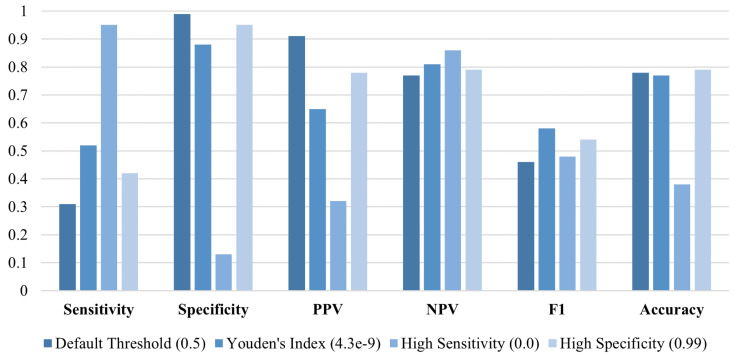
Results of PatchCTG model pretrained on a subset of cases recorded 3–7 days before delivery, followed by finetuning and testing on cases recorded up to 2 days prior to delivery (AUC = 0.75).

To further validate the generalizability of PatchCTG, we employed a pretraining and finetuning strategy, aimed at adapting the model to new temporal subsets of the data. The model was first pretrained using CTG signals from cases recorded 3 to 7 days before delivery and then finetuned and evaluated on a subset with cases recorded within 2 days before delivery (Figure 5 and Figure 6). This approach aimed to assess how pretraining on a temporally broader subset could enhance prediction performance on cases closer to delivery. During the pretraining phase, the PatchCTG model achieved an AUC of 0.73 when trained solely on the subset of cases recorded 3 to 7 days before delivery. The metrics at various thresholds indicated that while specificity remained high, at 99% for the default threshold, sensitivity was relatively lower at 24%, reflecting the need for further adaptation in order for the model to predict outcomes more accurately when applied to a different temporal window.

Following pretraining, the model was finetuned using CTG signals from the APO cohort recorded within 2 days before delivery, with the results presented in Figure 6. This finetuning resulted in a performance boost, with the AUC improving to 0.75. This showed that adapting the model to the specific temporal characteristics of the test cohort improved its predictive accuracy. Specifically, its sensitivity increased from 31% to 52% at the Youden’s index threshold, while its specificity remained high, at 88%, showing that the model successfully adapted to temporal shifts in the data.

### 4.4. Benchmark Evaluation

**Figure 7:**
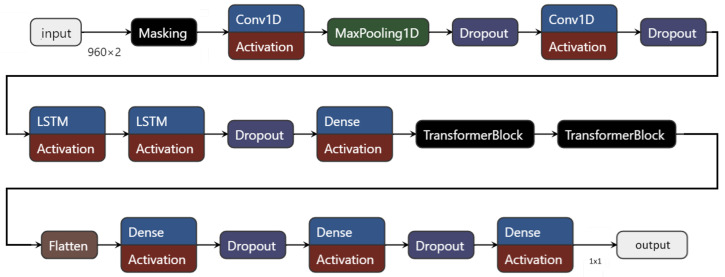
Optimal hybrid deep learning model identified through hyperparameter tuning for benchmark comparison with PatchCTG.

Our benchmark comparison evaluates the performance of PatchCTG relative to the DR algorithm and a hybrid deep learning model for classifying antepartum CTG data into adverse and normal outcomes. The hybrid deep learning model was optimised via an extensive hyperparameter tuning process, which explored variations in its CNN, LSTM, and transformer layers to identify the most effective architecture for CTG classification. The hyperparameters for the CNN layers included up to three layers with varying filters, kernel sizes, L2 regularisation, activation functions, and dropout rates. The LSTM layers were tuned for unit count, regularisation, and dropout, while transformer blocks were tested with different embedding dimensions, feed-forward dimensions, attention heads, and block counts. The optimal architecture (shown in Figure 7) identified through this process included two CNN layers, three LSTM layers, and two transformer blocks, achieving an AUC of 0.74. This model achieved a sensitivity of 72% and specificity of 64% at the Youden’s index threshold. In comparison, the traditional Dawes–Redman (DR) algorithm attained an AUC of 0.67, reflecting a lower discriminatory power than both the CNN-LSTM-Transformer model and PatchCTG. The DR algorithm demonstrated a high specificity (90.7%) but low sensitivity (18.2%), correctly identifying the majority of normal outcomes but showing a limited capacity to detect adverse outcomes. In contrast, PatchCTG achieved the highest AUC of 0.77, outperforming both the CNN-LSTM-Transformer model and the DR algorithm. At the Youden’s index threshold, PatchCTG attained a balanced sensitivity of 57% and specificity of 88%. PatchCTG’s high AUC demonstrates its effective handling of both the local and global temporal dependencies in CTG signals, attributed to its patch-based segmentation and self-attention mechanisms, which enable it to adapt to the diverse temporal patterns and signal variabilities inherent in CTG data.

## 5. Discussion

PatchCTG demonstrated a robust performance, with an AUC of 0.77, highlighting its capacity to accurately classify CTG recordings as adverse or normal in the antepartum setting. Compared to traditional visual CTG interpretation, which suffers from high variability (with an inter-observer agreement as low as 29%) and a high false positive rate (60%), PatchCTG provides a more consistent, objective evaluation. It also outperforms the Dawes–Redman system [1] and machine learning approaches [15,17]. By leveraging self-attention mechanisms and patch-based feature extraction, PatchCTG addresses long-standing issues in CTG interpretation, including inter-observer variability and a limited predictive capability for adverse outcomes. This consistency is critical given the limitations of prior models, which have lower specificity and varying sensitivities, often requiring complex feature engineering to capture the nuanced temporal patterns in FHR signals [16,18]. The integration of patch-based segmentation and self-attention mechanisms in PatchCTG represents a significant advancement in CTG analysis, drawing on transformer architectures that have shown promise in other medical time series applications [9]. By segmenting CTG signals into patches and applying instance normalisation and channel-independent processing, PatchCTG efficiently captures both local and global temporal dependencies, which are essential for interpreting the physiological dynamics in FHR and uterine contraction signals. Unlike convolutional models [20,24], which excel in extracting spatial features but can struggle with long-range dependencies, PatchCTG leverages a self-attention mechanism to dynamically adjust its focus across signal patches, enhancing its ability to detect subtle patterns associated with adverse outcomes.

Evaluating PatchCTG at different temporal thresholds demonstrated its generalisability across various intervals before delivery, with some reduction in performance for signals recorded closer to delivery. This degradation may reflect the increased variability and subtle changes in physiological patterns as delivery approaches, emphasising the potential value of incorporating additional clinical markers or features into the model to improve its prediction accuracy during these critical hours. Importantly, the ability of the model to leverage broader temporal data during pretraining, with finetuning on closer-to-delivery signals, illustrates an approach beneficial for clinical settings where data from different time windows may vary in availability and relevance. The performance of PatchCTG across different classification thresholds also underscores its adaptability to clinical priorities. By adjusting the threshold to increase sensitivity, the model can be tuned to minimise false negatives, which is essential in high-risk clinical scenarios where missing an adverse outcome could lead to severe consequences. Conversely, a high specificity threshold could help reduce unnecessary interventions when the priority is to avoid false positives. This flexibility makes PatchCTG a valuable tool for aiding clinical decision-making in fetal monitoring.

Benchmark comparisons further underscore the strong performance of PatchCTG relative to an optimized hybrid deep learning model and the DR algorithm. With an AUC of 0.77, PatchCTG outperformed the CNN-LSTM-Transformer model, which achieved an AUC of 0.73, and the Dawes–Redman algorithm, which had an AUC of 0.67. This comparison underscores the enhanced capability of PatchCTG to capture critical temporal dependencies while maintaining high predictive accuracy, particularly compared to the conventional methods, which exhibit lower specificity and sensitivity trade-offs. Overall, PatchCTG addresses the gaps identified in prior deep learning methods used for CTG analysis by efficiently capturing temporal dependencies, reducing subjectivity, and enabling adaptable outputs.

In clinical contexts, a sensitivity above 60% and specificity above 85% are typically considered practical targets for antepartum screening, balancing the need to detect adverse outcomes with the need to limit the occurrence of excessive false positives or unnecessary interventions [35]. To further support prediction close to delivery, incorporating clinical markers such as the maternal heart rate, obstetric risk factors (e.g., gestational diabetes, preeclampsia), or biochemical indicators (e.g., fetal lactate, pH) could provide important context. However, the inclusion of such features introduces additional challenges, including increased model complexity, the need for the reliable capture and integration of multimodal data, and potential risks of overfitting, particularly in settings with limited data diversity or infrastructure [36]. Future research should explore robust multimodal fusion strategies and deployment-aware workflows for integrating clinical and biomedical data into AI-enabled CTG systems, with a focus on enhancing predictive performance near delivery and validating model generalisability across diverse, real-world cohorts.

## 6. Conclusions

This study introduces PatchCTG, a transformer model explicitly designed for antepartum CTG-based fetal health monitoring, which overcomes the limitations of visual CTG interpretation, rule-based systems, and existing machine learning methods. Achieving an AUC of 0.77, PatchCTG outperformed the Dawes–Redman system (AUC of 0.67) and an optimised hybrid deep learning model (AUC of 0.73), demonstrating its superior ability to capture complex temporal dependencies and provide clinically relevant predictions. The ability of PatchCTG to capture complex local and global temporal dependencies, along with its adaptability across varying timeframes, positions it as a valuable tool for clinical application, offering greater reliability and objectivity than traditional methods. Its adaptable sensitivity and specificity thresholds further enhance its clinical utility, allowing for precision adjustments that prioritise sensitivity in high-risk cases or emphasise specificity to minimise unnecessary interventions. This flexibility, combined with the capacity of PatchCTG to generalise across different temporal windows, supports this being a robust approach to CTG interpretation that can help reduce the subjectivity common in manual assessments. While the performance of PatchCTG is promising, further enhancements could be achieved by incorporating additional data sources and clinical markers, particularly to improve its predictive accuracy closer to delivery. Future work will focus on expanding its clinical validation across diverse datasets and exploring the integration of multimodal inputs to enhance fetal health assessments and support more timely, informed clinical decisions.

## Data Availability

This study used the Oxford Maternity (OXMAT) dataset [12], a curated repository of CTG traces and maternal–neonatal health records. The dataset includes over 211,000 CTG recordings collected from more than 250,000 pregnancies between 1991 and 2024, providing extensive clinical data for model evaluation. Further details about the dataset are available in the paper on the OXMAT (https://arxiv.org/abs/2404.08024, accessed on 7 April 2025) and on the OXMAT website (https://www.oxdhl.com/resources, accessed on 7 April 2025).
